# Trend of SARS-Cov-2-Specific IgG Antibody Titers Among COVID-19 Pneumonia Patients: A Nine-Month Follow-Up

**DOI:** 10.7759/cureus.40860

**Published:** 2023-06-23

**Authors:** Manzoor Ahmad, Mohammad Muttahir Uddin, Abdul Ahad Wani, Ishrat Majeed, Ruquaya Aziz, Mubarak Naqash

**Affiliations:** 1 Department of General Medicine, SKIMS (Sher-i-Kashmir Institute of Medical Sciences) Medical College, Srinagar, IND; 2 Department of Community Medicine, Government Medical College (GMC), Srinagar, IND; 3 Department of Biochemistry, SKIMS (Sher-i-Kashmir Institute of Medical Sciences) Medical College, Srinagar, IND; 4 Department of General Medicine and Biochemistry, SKIMS (Sher-i-Kashmir Institute of Medical Sciences) Medical College, Srinagar, IND

**Keywords:** reverse-transcription polymerase chain reaction (rt-pcr) test, severe acute respiratory syndrome coronavirus 2 specific igg antibody titres, coronavirus disease 19 pneumonia, supplemental oxygen, prospective study, seropositivity rates, immunological memory

## Abstract

Introduction

Understanding the dynamics of anti-severe acute respiratory syndrome coronavirus 2 (SARS-CoV-2) immunoglobulin G (IgG) spike antibody titers after natural infection is important for understanding immunological memory. This longitudinal study was conducted to assess the trend in serum SARS-CoV-2 IgG spike antibody titers in a cohort of recovered cases up to nine months after SARS infection.

Materials and methods

We examined the neutralizing antibody response (IgG spike) in serum samples from a cohort of 86 SARS-CoV-2 quantitative polymerase chain reaction (qPCR)-confirmed infection, comprising cases having minor COVID-19 pneumonia and severity, which was determined by CT severity scores. Patients were enrolled in August/September 2020 and serum samples have been processed at one, three, six, and nine months. CT severity scores were rated between 1-25 and antibody titers≥ 1.4 were considered positive.

Results

The mean anti-SARS-CoV-2-specific IgG antibody titers at one month, three months, six months, and nine months were 22.02 ± 18.36, 14.62 ± 12.61, 8.93 ± 8.10, and 3.86 ± 5.70, respectively. The difference was statistically significant. The seropositivity rates (titer ≥1.4 IU) were 93.02%, 82.56%, 76.74%, and 58.14% at one, three, six, and nine months after infection, respectively. Cases with severe CT severity scores showed significantly higher mean antibody levels at all follow-up visits.

## Introduction

Globally, the severe acute respiratory syndrome coronavirus 2 (SARS-CoV-2) pandemic imparted substantial morbidity and mortality and has been responsible for significant disruption to routine activities and affected the quality of life [1,2]. The pandemic spread across the world at unprecedented speed due to vulnerable populations to the novel SARS-CoV-2 virus [3]. Initially, it is considered that the infection might develop immunity among the individuals and thus halts the spread of this pandemic, considering the dynamics of neutralizing antibodies in people infected with SARS-CoV-2 [4]. At present, there are only limited data on the dynamics of neutralizing antibodies after subsequent recovery from SARS-CoV-2 [5]. In most acute viral infections, the neutralizing antibodies rise promptly after infection due to a burst of short-lived anti-body-secreting cells and then fall from this peak before reaching a stable plateau that can be sustained by long-lived plasma and memory B cells for years to decade’s cells [6]. This trend has been observed in most viral infections and in other seasonal coronaviruses [7-9]. Understanding the dynamics of SARS-CoV-2 immunoglobulin G (IgG)-spike antibody titers in recovered cases can shed light on the duration of humoral immunity induced by natural infection and the influence of disease severity on antibody titers. The objectives of the current prospective study were to conduct a longitudinal evaluation of anti-SARS-CoV-2 IgG-S antibodies for a nine-month follow-up duration among patients hospitalized with SARS-CoV-2 pneumonia and to systematically illustrate predictors of the serological reaction besides SARS-CoV-2.

## Materials and methods

Study design and setting

This prospective study was conducted on patients who were discharged during the period from 24 August 2020 and 8 September 2020 and followed up for the next nine months and was performed in a tertiary care hospital, SKIMS Medical College, Srinagar, after getting approval from the ethical committee (ethical committee approval number - IEC/17/2020). This institute had been designated as a coronavirus disease 2019 (COVID-19) hospital during the pandemic. The Kashmir valley is a landlocked region in northern India, and the study hospital catered to patients from across the valley. The valley saw its first confirmed case in March 2020 after which new infections increased daily and the first spike in infections was observed in April and May 2020, when the daily new infections in the country reached around 3.5 lakh cases per day [10].

The average number of daily new infections during study recruitment was between 20,000 and 30,000 per day. The hospital provided both outpatient and inpatient services for confirmed COVID-19 patients. Patients requiring supplemental oxygen (SPO2 ≤ 94), those with comorbidity, or cases where the treating physician saw an increased risk of SARS-CoV-2 complications were hospitalized while the remainder were treated on an OPD basis [11]. The most common complications that warranted hospital admission despite normal oxygen saturation (SPO2) were those with cardiovascular disease, long-standing hypertension or diabetes, and COPD [12,13].

Participants

Study participants were selected from the admitted patients and the inclusion criteria as a positive reverse-transcription polymerase chain reaction (RT-PCR) test for SARS-CoV-2 patients aged more than 18 years and admitted to the hospital for at least 24 hours. To ensure follow-up for sample collection, only subjects residing within 5 km of the hospital have been enrolled in the study. Subjects showing a history of any immunocompromised condition, history of organ transplant, and persons living with HIV were excluded from the study [14].

Procedure

On the day of discharge, eligible cases were informed regarding the purpose and process of the research plan and were given the option to participate voluntarily. Trained health personnel conducted interviews and recorded responses on a pre-tested schedule. Contact details were recorded and participants were made aware of the schedule for blood sample collection. The first sample was collected between four and six weeks after the date of the first positive RT-PCR test for SARS-COV-2. Second, third, and fourth samples were collected at three months (± 1 week), six months (± 1 week), and nine months (± 1 week) after the date of the first positive test for SARS-COV-2. For sample collection, a trained phlebotomist collected 3-5 mL of venous blood from the antecubital vein under aseptic precautions into a red-top collection tube containing a clot activator. The tube was left vertical, untouched, for thirty minutes for the development of clots. Samples were then processed for centrifugation. Centrifuged samples were transported to a central laboratory for additional treatment and investigation [10]. Serum samples were then verified for the occurrence of SARS-CoV-2-specific IgG Spike antibodies by the Abbott SARS-CoV-2 IgG assay. Labeling was done for assay results equal to or above the cut-off index value of 1.4 as positive for SARS-CoV-2-specific IgG antibodies.

Variables

The primary outcome variable of interest was SARS-CoV-2-specific IgG antibodies and CT scan severity for pneumonia. Visual severity assessment of the chest CT has been categorized as Score 1 (less than 5% affected zone), Score 2 (5% to 25% affected zone), Score 3 (25% to 50% affected zone), Score 4 (50% to 75% affected zone), Score 5 (greater than 75% affected zone). This was done separately for every five lobes and a CT severity score with a maximum value of 25 was assigned. This variable was converted into three classifications: Mild (score less than 7), Moderate (score 7-18), and Severe (score greater than 18).

Statistical analysis

Descriptive statistics were calculated for categorical variables and represented as numbers and percentages. The normality of the data was confirmed by Kolmogorov-Smirnov/Shapiro-Wilk tests. If the continuous variables fit a normal distribution, the mean and standard deviation has been considered, and if not, the median and 25th to 75th percentiles (interquartile range) were used. Categorical parameters were associated with Pearson χ2 or Fisher's exact tests. The Mann-Whitney U test was used for comparing two groups and the Kruskal-Wallis test for more than two groups since the quantitative antibody levels were not normally distributed. The data was processed using Stata V.15 (StataCorp, College Station, TX).

## Results

Demographic and clinical characteristics

**Figure 1:**
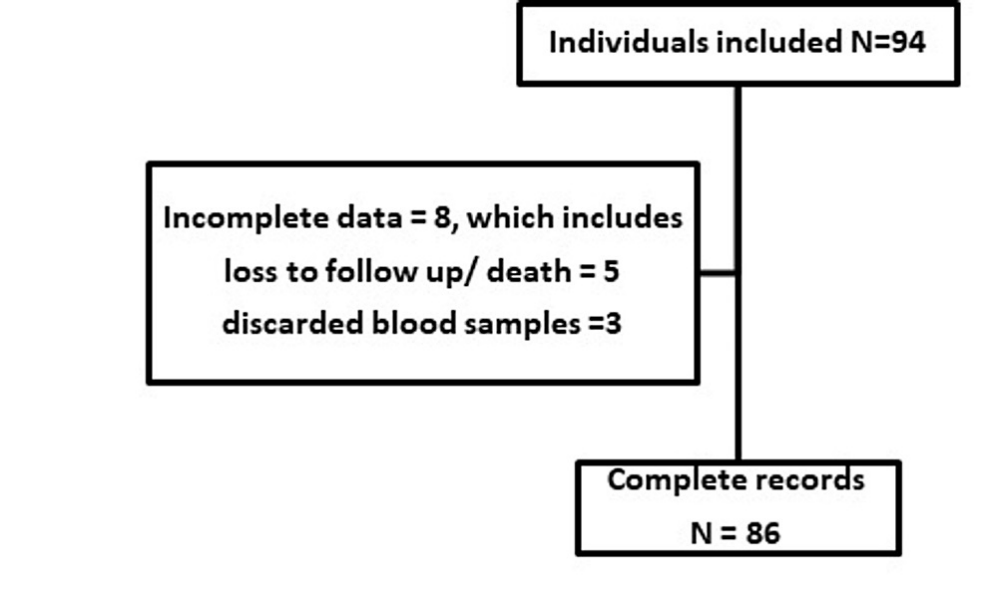
Flowchart depicting the number of patients at each stage

Our study enrolled 94 patients between 24 August 2020 and 8 September 2020 during discharge from the hospital. Eighty-six patients who were hospitalized with COVID-19 pneumonia were included in the final investigation (Figure 1).

The demographic and experimental characteristics of 86 recovered individuals with laboratory-confirmed COVID-19 are presented in Table 1. Of the participants, 23 (26.74%) were in the 40 to 49 age group and the same number belonged to the age group of 60-69. Subjects aged ≥ 70 years comprised 17 (19.77%) participants. Males comprised 57 (66.28%) participants. All subjects reported at least one symptom. The most frequently reported symptoms were fatigue, reported by 79 (91.86%) of the participants, followed by upper respiratory symptoms in 64 (74.42%), fever in 60 (69.77%), and myalgia/arthralgia with 55 (63.95%) symptoms. Cough, chills, and dyspnoea were reported by nearly half of the participants, as was anosmia. Of the patients, 42 (48.84%) were hypertensive and 29 (33.72%) were diabetic. Almost one-fourth (24.42%) had evidence of cardiovascular disease while four (4.65%) patients had chronic kidney disease. Chronic obstructive pulmonary disease (COPD) was present in seven (8.14%) patients. The median length of hospital stay is 10 days with an interquartile range (IQR) of 9-14 days. The mean SPO2 with room air on admission was 84.55 ± 4.96. At hospital admission, nearly half of the participants (48.84%, 42 out of 86) had moderately severe COVID-19 pneumonia (CT severity score between 7 and 18), and 33.72% (29 out of 86) had a CT scan severity score in the severe category (CT score 18) and the remainder (17.44%, 15 of 86) had a CT severity score in the mild category (CT score <7).

**Table 1:** Demographic and clinical profile of participants

Gender	Frequency	%
Male	57	66.28
Female	29	33.72
Age Mean ± SD (years)	54.59 ± 14.77	
Age		
18 - 39 Years	11	12.79
40 - 49 years	23	26.74
50 - 59 years	12	13.95
60 - 69 years	23	26.74
≥ 70 years	17	19.77
Symptoms at admission		
Fever	60	69.77
Cough	46	53.49
chills	45	52.33
Dyspnoea	47	54.65
Fatigue	79	91.86
Myalgia/Arthralgia	55	63.95
Hemoptysis	9	10.47
Sore throat	27	31.40
Rhino Rea	10	11.63
Diarrhea	19	22.09
Vomiting/ Nausea	17	19.77
Chest pain	29	33.72
Headache	31	36.05
Anosmia	43	50.00
Comorbidities		
Hypertension	42	48.84
Diabetes	29	33.72
CVD	21	24.42
CKD	4	4.65
COPD	7	8.14
Hypothyroidism	18	20.93
Any other	12	13.95
CT severity score		
Mild (score <7)	15	17.44
Moderate (score 7 – 18)	42	48.84
Severe ( score > 18)	29	33.72
SPO2 at admission (room air) Mean ± SD	84.55 ± 4.96	
No. of days admitted Median ( IQR)	10 (9 - 14)	
No. of days admitted (Range in days)	2-41 Days	

The mean SARS-CoV-2-specific IgG antibody titers at one, three, six, and nine months were 22.02 ± 18.36, 14.62 ± 12.61, 8.93 ± 8.10, and 3.86 ± 5.70. Table 2 depicts the trend in antibody levels for all the patients over the nine-month follow-up period (p=0.0028).

**Table 2:** SARS-CoV-2-specific IgG antibody titers at one, three, six, and nine months

Duration	SARS-CoV-2-specific IgG antibody titers	P-value
One month	22.02 ± 18.36	0.0028
Three months	14.62 ± 12.61	
Six months	8.93 ± 8.10	
Nine months	3.86 ± 5.70	

There was a substantial decline in the proportion of cases that were seropositive at each follow-up visit. The percentage decreased from 93.02% to 58.14% at nine months after infection (Table 3).

**Table 3:** Trend of overall seroprevalence

	1 month	3 months	6 months	9 months
Seroprevalence (%)	93.02%	82.56%	76.74%	58.14%

**Figure 2:**
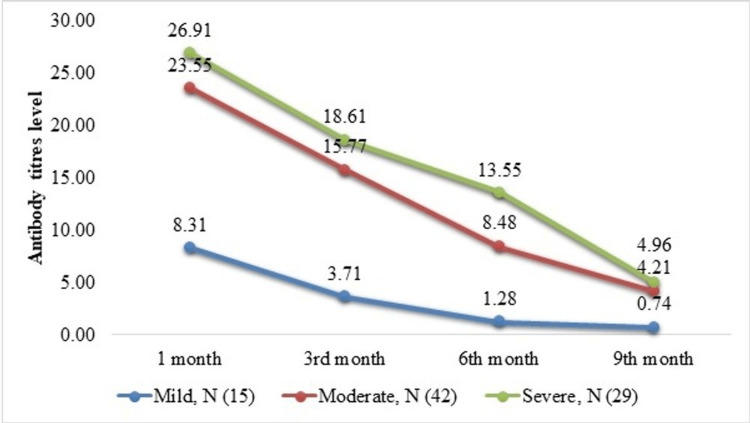
Mean IgG levels as per the severity of COVID-19 pneumonia The Kruskal–Wallis test was used to estimate differences between the three groups at each follow-up. At one month, p = 0.0037, at three months, p = 0.0004, at six months, p <0.000, at nine months, p = 0.0554

The antibody levels at all follow-up points were greater among cases with a severe CT severity score than among persons with moderate and mild CT severity scores. The antibody titers also decreased significantly in all three groups (Figure 2).

Our observation reveals a significant association between CT severity score at admission and serum IgG titers at one month, and that CT severity score is also significantly associated with serum IgG titers at all follow-up points. Additionally, the study found that a history of diabetes mellitus is negatively correlated with serum IgG titers at three, six, and nine months, but not at one month, indicating that antibody titers decrease faster among individuals with diabetes (Table 4).

**Table 4:** Linear regression for serum IgG titers at different follow-ups IgG: immunoglobulin G; CONS: coagulase-negative staphylococci (CONS)

Variables	Serum IgG at 1 month P>|t|	Serum IgG at 3 months P>|t|	Serum IgG at 6 months P>|t|	Serum IgG at 9 months P>|t|
Gender	0.193	0.000	0.005	0.054
Age	0.646	0.149	0.226	0.417
Days of admission	0.615	0.294	0.569	0.800
Hypertension	0.261	0.790	0.421	0.892
Diabetes	0.075	0.007	0.008	0.006
Fever at admission	0.838	0.857	0.931	0.245
SPO2 at admission	0.642	0.861	0.147	0.291
CT severity score	0.000	0.003	0.007	0.002
CONS	0.000	0.010	0.275	0.310

## Discussion

The immune response to natural SARS-CoV-2 infections might have an important impact on population-level transmission. We used a prospective design in patients discharged in August/September 2020 who were followed up for the next nine months and had serum IgG antibodies to SARS-CoV-2 estimated at one, three, six, and nine months. Patients were admitted from a single tertiary-care hospital in the Kashmir valley that was accepting SARS-CoV-2 pneumonia patients who required supplemental oxygen or assisted ventilation. In addition, some patients who did not otherwise require supplemental oxygen were admitted because of their comorbidities. All adults over the age of 18 were eligible, although those with an immunocompromising condition were excluded from the study. We flagged assay observations equal to or beyond the cut-off index value of 1.4 as positive for SARS-CoV-2-specific IgG antibodies. Chest CT scan severity was scored from 0-25 [15]. This variable was converted into three categories: Mild (score less than 7), Moderate (score 7-18), and Severe (score greater than 18) [16].

We enrolled 94 patients, 86 of whom were included in the final analysis. Males comprised 57 participants and the subjects had a mean age of 54.59 to 14.77 years. Almost half of the subjects were hypertensive and one-third were diabetic. Twenty-one subjects had cardiovascular disease and seven had COPD. On admission, the most common symptoms were fatigue (91.86%), fever (69.77%), cough (53.49%), dyspnoea (54.65%), and chills (52.33%). Anosmia was present in 50% of the cases. Most subjects (42 of 86, 48.84%) had a moderate CT severity score and 29 (33.72%) subjects had a severe CT severity score.

Mean anti-SARS-CoV-2-specific IgG antibody titers at one, three, six, and nine months were 22.02 ± 18.36, 14.62 ± 12.61, 8.93 ± 8.10, and 3.86 ± 5.70 IU/ml, respectively. The decrease was statistically significant. Several current pieces of research also ensure that IgG antibodies against SARS-CoV-2 decrease many-fold within the first few months after infection [17,18].

It suggests that the humoral reaction to SARS-CoV-2 is comparable to further acute viral infections. The seropositivity rate (titer ≥1.4 IU) was 93.02%, 82.56%, 76.74%, and 58.14% at one, three, six, and nine months after infection. The decrease in seroprevalence is consistent with several other studies, which found that there is a significant decrease in infections at six months [18,19]. The results of our study differ from a study conducted among healthcare professionals, in which almost all participants remained seropositive at 12 months [20]. This might be due to repeated subclinical infections/exposure to the SARS-CoV-2 virus among healthcare providers who are at higher threat of occupational exposure. Higher CT severity at admission was significantly associated with higher antibody titers at one, three, six, and nine months. Antibody titers were significantly higher in all intervals with severe CT scores. This suggests that a higher degree of lung involvement leads to higher antibody titers that persist even nine months after infection. This is consistent with multiple previous studies in which antibody titers were higher at six months for subjects with severe and moderate disease than in those with mild disease [21,22]. On multivariate analysis, a history of diabetes mellitus was negatively correlated with serum IgG titers at three, six, and nine months but not at one month, suggesting that antibody titers decline more rapidly in individuals with diabetes. This is consistent with previous studies that found a more rapid decline in humoral immunity in diabetics.

The main limitation of the study was we don't have the data since the study was performed after April 2020 and at that time, the majority of the patients were discharged at that time with resolved symptoms. Further, the study also lacks clinical and serological data among the patients who had died, so we are unable to predict the association between serological characteristics and survival. 

## Conclusions

We conclude that anti-SARS-CoV-2 IgG-S antibody titers at follow-up among recovered patients are greater in cases with a higher CT severity score at admission. The titers gradually decline in all patients and around 58% of patients are still seropositive at nine months after infection. Since almost half of the patients don’t have the requisite protective antibody titers after nine months, it is recommended that recovered patients be vaccinated for COVID-19 to boost antibody response and to protect from re-infection.
